# De novo *CTBP1* variant is associated with decreased mitochondrial respiratory chain activities

**DOI:** 10.1212/NXG.0000000000000187

**Published:** 2017-09-22

**Authors:** Ewen W. Sommerville, Charlotte L. Alston, Angela Pyle, Langping He, Gavin Falkous, Karen Naismith, Patrick F. Chinnery, Robert McFarland, Robert W. Taylor

**Affiliations:** From the Wellcome Centre for Mitochondrial Research (E.W.S., C.L.A., L.H., G.F., R.M., R.W.T.), Institute of Neuroscience, Newcastle University, United Kingdom; Department of Molecular and Human Genetics (E.W.S.), Baylor College of Medicine, Houston, TX; Wellcome Centre for Mitochondrial Research (A.P.), Institute of Genetic Medicine, Newcastle University; Armistead Child Development Centre (K.N.), Kings Cross Hospital, Dundee, Scotland; Department of Clinical Neurosciences (P.F.C.), School of Clinical Medicine, University of Cambridge; and MRC Mitochondrial Biology Unit (P.F.C.), University of Cambridge, United Kingdom.

## Abstract

**Objective:**

To determine the genetic etiology of a young woman presenting an early-onset, progressive neurodegenerative disorder with evidence of decreased mitochondrial complex I and IV activities in skeletal muscle suggestive of a mitochondrial disorder.

**Methods:**

A case report including diagnostic workup, whole-exome sequencing of the affected patient, filtering, and prioritization of candidate variants assuming a suspected autosomal recessive mitochondrial disorder and segregation studies.

**Results:**

After excluding candidate variants for an autosomal recessive mitochondrial disorder, re-evaluation of rare and novel heterozygous variants identified a recently reported, recurrent pathogenic heterozygous *CTBP1* missense change (c.991C>T, p.Arg331Trp), which was confirmed to have arisen de novo.

**Conclusions:**

We report the fifth known patient harboring a recurrent pathogenic de novo c.991C>T p.(Arg331Trp) *CTBP1* variant, who was initially suspected to have an autosomal recessive mitochondrial disorder. Inheritance of suspected early-onset mitochondrial disease could wrongly be assumed to be autosomal recessive. Hence, this warrants continued re-evaluation of rare and novel heterozygous variants in patients with apparently unsolved suspected mitochondrial disease investigated using next-generation sequencing.

Mitochondrial respiratory chain disorders are among the most common early-onset metabolic disorders with an estimated minimum point prevalence of 1 in 5,000 live births.^1^ Early-onset respiratory chain disorders are characterized by extreme clinical, molecular, and genetic heterogeneity due to pathogenic mitochondrial DNA (mtDNA) or nuclear gene variants affecting proteins essential for mitochondrial functions. The diagnosis of suspected mendelian mitochondrial disorders is especially challenging given that an estimated 1,200 mitochondrial proteins are encoded by the nuclear genome. Whole-exome sequencing (WES) has been widely used as a cost-effective tool for the diagnosis of suspected mendelian, early-onset mitochondrial respiratory chain disorders.^2–5^ However, secondary respiratory chain defects in skeletal muscle have also been described in other neuromuscular and neurologic disorders due to pathogenic variants of genes encoding nonmitochondrial proteins,^6,7^ providing additional challenges to candidate variant prioritization.

We describe a young woman presenting an early-onset, progressive neurodegenerative disorder with decreased mitochondrial complex I and IV activities in skeletal muscle. Using WES, we identified a recurrent pathogenic de novo heterozygous *CTBP1* variant previously associated with an early-onset neurologic disorder.^8^ We highlight the challenges of variant prioritization in a patient who was initially suspected to have an autosomal recessive mitochondrial disorder.

## METHODS

### Muscle histopathological, biochemical, and genetic studies.

Diagnostic muscle biopsy was performed according to standard procedures. Muscle biopsy was subjected to cytochrome *c* oxidase (COX) and succinate dehydrogenase (SDH) histochemical reactions.^9^ Mitochondrial respiratory chain complex activities (complexes I-IV) relative to citrate synthase were measured in the skeletal muscle homogenate as previously described.^10^ Whole mitochondrial genome sequencing was performed to exclude pathogenic point mutations and small insertions or deletions, while diagnostic quantitative real-time PCR and long-range PCR assays were performed for mtDNA quantification and detection of rearrangements, according to standard protocols.

### Whole-exome sequencing, analysis, and interpretation.

Exome capture was attained using the Illumina TruSeq 62 Mb capture kit, sequenced using the Illumina HiSeq 2000 system in 100 base pair (bp) reads, and aligned to the human reference genome (UCSC hg19). Variants with a minor allele frequency ≥0.01 (1%) from 378 in-house controls and external variant databases including the Exome Aggregation Consortium, National Heart, Lung, and Blood Institute Exome Variant Server (Exome Sequencing Project), and 1000 Genomes Project were excluded. Autosomal recessive (compound heterozygous or homozygous) variants in nuclear genes encoding mitochondrial-localized proteins were prioritized. Autosomal recessive variants of all nuclear genes were next examined, followed by examination of rare and novel heterozygous variants. PolyPhen-2, SIFT, and Align Grantham Variation with Grantham Deviation (GVGD) were used to assess the pathogenicity of candidate variants.

### Sanger sequencing validation.

Candidate variants were validated by Sanger sequencing using standard protocols.^2^ Custom forward and reverse primers were designed for all exons and intronic regions of *EARS2* (NM_001083614) and exon 9 of *CTBP1* (NM_001012614).

## RESULTS

### Case report.

This female patient was born to nonconsanguineous parents following a normal pregnancy. Very early development was considered normal though, while the patient was able to sit independently at 8 months, she disliked being placed prone and never acquired the ability to crawl. She did manage to walk with hand-holding support at the age of 16 months but generalized hypotonia and weakness meant that independent ambulation was never achieved and she started to use a wheelchair. Fine motor skills were variable in that she could turn the pages of a book and hold a crayon, but had difficulty drawing. Language acquisition was limited to a few words in early life and these were lost by the age of 4 years. Scoliosis, requiring corrective surgery, had developed by 10 years, and at 14 years, she had pneumonia with cardiorespiratory arrest. Following successful resuscitation, she was notably weaker and dysphagic, becoming dependent on a percutaneous endoscopic gastrostomy tube for feeding. Brain MRI about 2 years prior to this event had revealed mild cerebellar and brainstem atrophy, but no neuroimaging was performed afterward. Clinical examination, at the age of 16 years, revealed a young woman with sunken eyes and thin tapering fingers. Movements of her limbs and face were infrequent and slow. Lower limbs were cold and edematous with livedo reticularis of the overlying skin. Although her muscle tone was generally low, she had increased tone at the elbows, wrists, knees, and ankles with contractures of the wrists and elbows. There was a marked spinal scoliosis, and muscle mass was generally decreased. At 16 years of age, she died of acute respiratory failure.

Her family history was unremarkable for neurologic disorders; she had 1 unaffected younger sibling. Diagnostic Sanger sequencing of *PLA2G6* (NM_003560) and *APTX* (NM_001195251) performed at another center was negative for pathogenic or likely pathogenic variants.

A skeletal muscle biopsy showed histopathologic abnormalities including variation in muscle fiber size, occasional internal nuclei, and evidence of denervation atrophy. These were accompanied by increasing accumulation of fibroadipose connective tissue and patchy lipid accumulation within the muscle, with excess lipid also surrounding the muscle. Oxidative enzyme histochemistry showed patchy loss of COX activity and clumped SDH reactivity; unfortunately, sequential COX-SDH histochemistry was not performed. Biochemical analysis of mitochondrial respiratory chain activities showed decreased complex I (57% of control) and IV (35% of control) activities, with sparing of the complex II activity (figure, A). Based on these findings, whole mitochondrial genome sequencing was performed but failed to detect known pathogenic or likely pathogenic variants. Quantitative real-time PCR and long-range PCR assays also excluded mtDNA depletion and mtDNA rearrangements.

**Figure:**
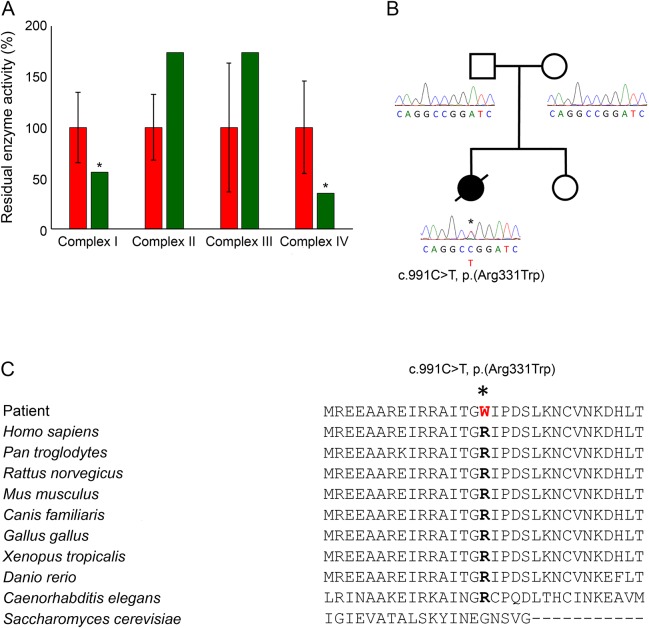
Biochemical and molecular genetic characterization of the de novo c.991C>T p.(Arg331Trp) *CTBP1* variant (A) Mitochondrial respiratory chain activities normalized to citrate synthase for complexes I-IV in patient's skeletal muscle (green) compared with controls (red). Asterisk denotes decreased complex I and IV activities. (B) Family pedigree and Sanger sequencing confirming that the c.991C>T p.(Arg331Trp) variant arose de novo. (C) Multiple sequence alignment demonstrating high conservation of the Arg331 residue.

### Whole-exome sequencing analyses.

Informed parental consent was obtained, and WES was performed. Anticipating a possible autosomal recessive mitochondrial disorder, nuclear genes encoding mitochondrial-localized proteins were prioritized. However, no likely candidate variants were identified, and inspection of all rare autosomal recessive variants did not reveal persuasive candidates. A rare heterozygous missense variant (c.452A>G, p.Lys151Arg) in *EARS2* (NM_001083614) encoding mitochondrial glutamyl-transfer RNA synthetase was identified; recessive pathogenic *EARS2* variants cause the mitochondrial disorder leukoencephalopathy with thalamus and brainstem involvement and high lactate.^11^ However, the c.452A>G p.(Lys151Arg) variant was not previously reported as pathogenic. In addition, Sanger sequencing of all exons and intronic regions of *EARS2* did not identify a second candidate variant. Examination of WES read coverage also did not disclose copy number variants or rearrangements involving *EARS2*.

Inspection of rare and novel heterozygous variants that were predicted to be damaging to protein function revealed a c.991C>T p.(Arg331Trp) missense change in *CTBP1* (NM_001012614.1). Following the report of 4 patients sharing the identical heterozygous *CTBP1* variant that arose de novo in each case,^8^ Sanger sequencing with extracted blood DNA from our patient and her parents confirmed that the c.991C>T p.(Arg331Trp) variant had arisen de novo or 1 parent was low level mosaic (figure, B). The c.991C>T p.(Arg331Trp) variant was absent from our in-house exomes and external databases, affected a highly conserved residue, and was predicted to be deleterious by PolyPhen-2, SIFT, and Align GVGD (figure, C).

## DISCUSSION

We performed WES of a woman presenting an early-onset, progressive neurodegenerative disorder and suggested mitochondrial etiology on the basis of decreased respiratory chain activities of complexes I and IV, with sparing of the complex II activity, in a muscle biopsy. Suspecting an autosomal recessive disorder of mitochondrial translation, filtering and interpretation of variants in nuclear genes encoding mitochondrially targeted proteins failed to identify likely candidates. Only after the evaluation of rare and novel heterozygous variants was a previously reported pathogenic c.991C>T p.(Arg331Trp) *CTBP1* variant identified and confirmed to have arisen de novo. All 4 previously reported patients presented with comparable phenotypes of hypotonia, ataxia, severe developmental delay, intellectual disability, and problems with weight gain.^8^ Similarly, our patient had generally low muscle tone, developmental regression, severe intellectual disability, and was nonambulatory. Cerebellar atrophy was also noted in our patient and 2 reported patients. Of interest, all previously described patients also had striking tooth enamel defects manifesting as hypoplasia or discoloration. By contrast, our patient had normal teeth on clinical examination, although there was no comment on the enamel.

*CTBP1* encodes C-terminal binding protein 1 (CtBP1), a transcriptional corepressor first identified as an E1A oncogene binding protein,^12^ but has also been shown to associate with DNA-specific repressors during human development and tumorigenesis,^13,14^ thereby regulating gene expression and DNA repair. Although CtBP1 does not localize to mitochondria, it has been identified as a key regulator of mitochondrial function and morphology.^15^ Knockout of *Ctbp1* in mouse embryonic fibroblasts (MEFs) resulted in elongated mitochondria, swollen cristae, and decreased cellular adenosine triphosphate (ATP), oxygen consumption, and mitochondrial membrane potential.^15^ Altered mitochondrial function and morphology were attributed to transcriptional repression of Bcl-2-associated X protein (*Bax*) by CtBP1. *BAX* has been demonstrated to be essential for Drp1-dependent fission,^16^ while also regulating mitochondrial-dependent apoptosis in response to glucose deprivation, for which its expression is modulated by association of CtBP1 with the *BAX* promotor.^15^ When *Bax* and *Ctbp1* were knocked out together in MEFs, this led to increased ATP production, oxygen consumption rate, and membrane potential, confirming a role for CtBP1 in maintaining mitochondrial functions.

The de novo c.991C>T p.(Arg331Trp) *CTBP1* variant occurs in the PLDLS C-terminal binding cleft, which is required for the recruitment of DNA-binding factors and components of the CtBP1 corepressor complex.^17^ Decreased complex I and IV activities in skeletal muscle of our patient suggests that *BAX* expression is altered by mutated CtBP1, with the c.991C>T p.(Arg331Trp) variant acting in a dominant-negative mechanism to disrupt mitochondrial function. CtBP1 may also regulate the expression of additional nuclear genes involved in mitochondrial functions, including nuclear genes encoding essential mitochondrial protein synthesis machinery. The original description of 4 patients with a recurrent de novo *CTBP1* variant did not report respiratory chain defects in skeletal muscle, although one patient was investigated for “pathogenic mitochondrial mutations”.^8^ As yet, it is unclear whether mitochondrial dysfunction contributes to the pathogenesis of this disorder or is a secondary consequence. Therefore, future studies of mitochondrial function and morphology in tissue from affected patients could provide further insights into the pathologic mechanisms involved.

Pathogenic de novo heterozygous variants have been widely associated with early-onset phenotypes that include autism and epileptic encephalopathies.^18,19^ By contrast, reported de novo variants associated with early-onset mitochondrial disorders are rare. Recently, 5 patients sharing a de novo c.1582C>T p.(Arg528Trp) *ATAD3A* variant with global developmental delay, hypotonia, optic atrophy, axonal neuropathy, and hypertrophic cardiomyopathy were identified.^20^ In addition, de novo c.239G>A p.(Arg80His) and c.703C>G p.(Arg235Gly) *SLC25A4* variants were reported in 7 patients with neonatal-onset respiratory insufficiency and severe mtDNA depletion in skeletal muscle.^21^ Candidate variant filtering strategies of early-onset mitochondrial disorders investigated using next-generation sequencing have typically prioritized autosomal recessive or X-linked variants in nuclear genes encoding mitochondrial-localized proteins.^2–5^ Although the diagnostic yield of early-onset mitochondrial disorders using next-generation sequencing can be as high as 60%, there remains a considerable proportion of cases without a diagnosis. Similarly, we initially prioritized autosomal recessive candidate variants based on the decreased complex I and IV activities in patient's skeletal muscle and an unremarkable family history. Although the respiratory chain defects due to the pathogenic *CTBP1* variant are a likely secondary consequence, our experience suggests that this by no means precludes the identification of pathogenic variants in nonmitochondrial disease genes or an autosomal dominant etiology.

The identification of a recurrent pathogenic de novo c.991C>T p.(Arg331Trp) *CTBP1* variant using WES in a patient with decreased complex I and IV activities serves to caution that inheritance of suspected early-onset mitochondrial disorders could wrongly be assumed autosomal recessive. Furthermore, possible identification of patients with respiratory chain defects due to variants involving “non-classical mitochondrial disease” genes should not be overlooked.^6,7^ Our observations suggest that pathogenic de novo heterozygous variants could be under-recognized in suspected early-onset mitochondrial disease, emphasizing the need to evaluate heterozygous variants with segregation studies in apparently unsolved and prospective cases.

## GLOSSARY

ATP: adenosine triphosphate
COX: cytochrome *c* oxidase
GVGD: Grantham Variation with Grantham Deviation
MEF: mouse embryonic fibroblast
mtDNA: mitochondrial DNA
SDH: succinate dehydrogenase
WES: whole-exome sequencing
